# It is the habit not the handle that affects tooth brushing - a randomised counterbalanced cross over study with young and healthy adults

**DOI:** 10.1186/s12903-024-04538-6

**Published:** 2024-07-02

**Authors:** Renate Deinzer, Zdenka Eidenhardt, Keywan Sohrabi, Manuel Stenger, Dominik Kraft, Bernhard Sick, Franz Götz-Hahn, Carlotta Bottenbruch, Nils Berneburg, Ulrike Weik

**Affiliations:** 1 Department of Medicine, Justus-Liebig-University Giessen, Klinikstr. 29, Giessen, 35392 Germany; 2 Faculty of Health Sciences, University of Applied Sciences Giessen, Ostanlage 45, Giessen, 35390 Germany; 3 Intelligent Embedded Systems, University of Kassel, Wilhelmshöher Allee 73, Kassel, 34121 Germany

**Keywords:** Toothbrushing, Oral hygiene, Ergonomics, Dental plaque, Self-assessment, Manual toothbrush, Toothbrush handle, Toothbrushing performance, Plaque removal

## Abstract

**Objective:**

To assess the effect of the toothbrush handle on video-observed toothbrushing behaviour and toothbrushing effectiveness.

**Methods:**

This is a randomized counterbalanced cross-over study. *N* = 50 university students and employees brushed their teeth at two occasions, one week apart, using either a commercial ergonomically designed manual toothbrush (MT) or Brushalyze V1 (BV1), a manual toothbrush with a thick cylindrical handle without any specific ergonomic features. Brushing behaviour was video-analysed. Plaque was assessed at the second occasion immediately after brushing. Participants also rated their self-perceived oral cleanliness and directly compared the two brushes regarding their handling and compared them to the brushed they used at home.

**Results:**

The study participants found the BV1 significantly more cumbersome than the M1 or their brush at home. (*p* < 0.05). However, correlation analyses revealed a strong consistency of brushing behavior with the two brushes (0.71 < *r* < 0.91). Means differed only slightly (all d < 0.36). These differences became statistically significant only for the brushing time at inner surfaces (d = 0.31 *p* = 0.03) and horizontal movements at inner surfaces (d = 0.35, *p* = 0.02). Plaque levels at the gingival margins did not differ while slightly more plaque persisted at the more coronal aspects of the crown after brushing with BV1 (d = 0.592; *p* 0.042).

**Discussion:**

The results of the study indicate that the brushing handle does not play a major role in brushing behavior or brushing effectiveness.

**Supplementary Information:**

The online version contains supplementary material available at 10.1186/s12903-024-04538-6.

## Background

Most diseases of the teeth and periodontium could be prevented simply by toothbrushing, which should remove the plaque adhering to the teeth [1, 2]. The effectiveness of toothbrushing depends not only on factors such as method and frequency, but also on the design of the toothbrush [3]. Therefore, toothbrushes are constantly being developed to support and improve the mechanical removal of plaque. As a result, there are countless designs, shapes and types of toothbrushes on the market, each promising better cleaning efficiency and oral health benefits [4–6]. In terms of advancements, there are clear differences between manual and powered toothbrushes. In manual toothbrushes, the shape of the handle [7, 8] along with the brush head and the bristles [9] has undergone major changes. In powered toothbrushes, however, the motion of the brush head (vibrational or oscillation-rotational) and its speed (standard, sonic, ultra-sonic) have evolved the most [10]. Not surprisingly, the handle of powered toothbrushes was not substantially changed, as it also serves as a housing for the brush's batteries and accordingly it is subject to certain requirements in terms of its thickness and length. Depending on the model, the shape is somewhat more elliptical or cylindrical, but in any case the diameter of the handle is always larger than that of a manual toothbrush [7].

The design and texture of the toothbrush handle affect how well one can grip and control the toothbrush while brushing [11, 12]. Also, it has been noted that the handle influences comfort, compliance and muscle fatigue during toothbrushing [13–15]. Accordingly, studies with children or people with limited dexterity show that toothbrush handles with specialised features (i.e. larger or extended handles and adaptable designs to meet different needs) can make brushing easier [16–20]. There is thus an implicit assumption that an ergonomic handle improves the toothbrushing process itself and, consequently, also the cleanliness of the teeth. So far, the relationship between toothbrush handle and toothbrushing behaviour was only considered in terms of the outcome of the behaviour—i.e. plaque reduction. In these studies, the measured effects on plaque reduction were mostly rather small [13, 17, 18, 21–23]. However, the effects of different toothbrush handles on the toothbrushing process itself were not yet investigated.

The aim of the present study is therefore to examine the effects of the toothbrush handle on toothbrushing behaviour. In order to be able to draw conclusions about the behaviour with different handles, two genuinely different handles need to be compared. Thus, it was investigated whether brushing with a conventional ergonomic manual toothbrush differs from brushing with a larger and heavier toothbrush that has a thick cylindrical handle without any other ergonomic features. It was assessed whether the brush affected the toothbrushing process. Previous research indicates, that individual characteristics of toothbrushing behaviour remain stable over time [24]. It was therefore hypothesised that the handle of the brush would influence the extent to which certain behavioural parameters were displayed, while the position of the person in the group would remain fairly stable.

## Methods

The study is part of a larger research project called “Brushalyze—Understanding the tooth brushing process all along: New research device for multi-sensorial detection and intelligent analysis of tooth brushing”. (Deutsche Forschungsgemeinschaft (DFG) *–* Project number 448034414; https://gepris.dfg.de/gepris/projekt/448034414?language=en).

### Ethics & data privacy

In accordance with the principles of the Declaration of Helsinki, the Ethics Board of the Department of Medicine at the University of Giessen (No. 261/19) approved the study protocol. This trial is registered with the German Clinical Trials Registry (www. drks. de; DRKS-ID: DRKS00029698; date of registration: 18/07/2022). Every participant provided written informed consent. The data collection was pseudonymised (i.e. a personal code was created for each participant and the information about the person was stored under this code).

### Study design, independent variable and randomisations

The study was conducted in a counterbalanced cross-over design. The independent variable was brushing with one of two test toothbrushes. All participants had two appointments in the laboratory, one week apart, during which they brushed their teeth. They were randomly assigned to one of two sequences: test toothbrush 1 at the first appointment and test toothbrush 2 at the second appointment vs. test toothbrush 2 at the first appointment and test toothbrush 1 at the second appointment. For randomisation, boxes were set up for female and male participants, each containing ten identical opaque containers each filled with a slip of paper indicating group allocation. The boxes were filled until the calculated sample size was reached. While the participant was undergoing the first clinical examination (see the section "procedures"), the examiner responsible for the assessment of the behaviour (CB) phoned an assistant not involved in the study and informed about the participant's sex. The assistant blindly pulled a container jar out of the respective box, opened it and told CB the group membership (group 1 or group 2).

**Fig. 1:**
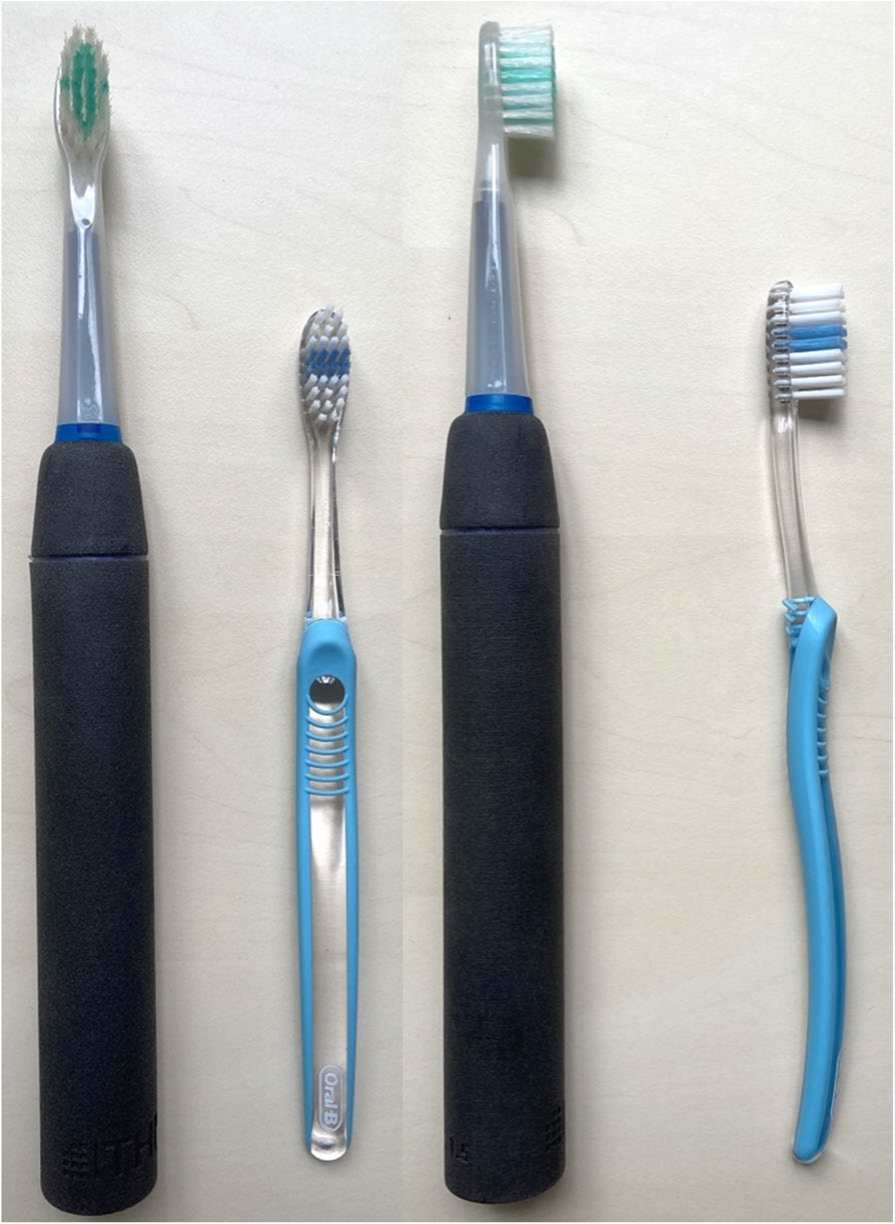
Test toothbrush 1: BV1 (black handle); Test toothbrush 2: MT (turquoise/transparent handle)
- *Test toothbrush 2 – Commercial manual toothbrush (MT).* The second tooth brush is a commercial brush with a brush head similar to that of the BV1 (see Fig. 1).^2^ In a pre-test, six members of the research team (2 dental and 4 non-dental) independently compared the shape and haptics of the bristle field of several commercial manual toothbrushes^3^ with that of the BV1 (see Fig. 1). They all voted for the selected brush as the one that most closely resembled the BV1 in this respect.

The following toothbrushes were used:

- *Test toothbrush 1 – Brushalyze-V1 (BV1)*. This toothbrush consists of a handle similar to that of an powered toothbrush. The handle is prepared for commercially available brush heads as used in sonic toothbrushes. The brush head used for current analysis has a flat bristle field.^1^ The bristles are arranged in an elliptical shape (see Fig. 1; for more details regarding the handle and its function within the Brushalyze project see Appendix).

### Participants

Participants were *N* = 50 students and employees of the Justus-Liebig-University of Giessen (Germany) who habitually brushed their teeth by a manual tooth brush (2/3 of tooth brushing events per week or more). They were recruited via a mailing that informed about the topic (toothbrushing), the basic procedure of the study and the financial compensation (30 €). Participants were eligible if they were 18—35 years old, had at least 20 natural teeth and a very good proficiency in German. In order to avoid participants bias, the following exclusion criteria were applied: training with a dental background; braces, fixed retainers, removable dentures; oral piercing or dental jewellery; pregnancy and breastfeeding; cognitive and physical limitations when brushing teeth (e.g. due to arm injury); professional tooth cleaning or tooth polishing in the past 4 weeks; acute or chronic diseases affecting the oral cavity (e.g. diabetes, HIV, eating disorders, herpes); medication for epilepsy, heart disease or immunosuppression; former participation in a tooth-brushing study of the institute.

### Procedures

The examinations took place between August and September 2022 at the Institute of Medical Psychology, Justus-Liebig-University of Giessen. To ensure the safety of the participants and the personnel (CB and NB), all persons involved in the study performed a rapid SARS-CoV-2 antigen test on all examination days. All experimenters interacted with the participants in a fully standardised manner by using always the same words and explanations. They were not allowed to talk to each other about the test persons and the observations they had made about them. All appointments with the participants were individual appointments with only one participant at a time.

*First appointment*: CB welcomed the participants and informed them in detail about the study procedure but kept them blind with regard to the precise research hypotheses. The participants read through the study consent form and then gave their written consent. Afterwards they went to the dental examination room where NB assessed the number of missing teeth, the papillary bleeding and dental status (DMF-T) of the participants. Then they entered an observation lab equipped with remote cameras and an intercom. There CB had prepared the randomly assigned condition (brushing with BV1 or MT). CB placed the participants in front of a washbasin and a tablet computer with a front-facing camera. With its camera, the tablet served as a mirror for the participants and it also allowed the toothbrushing procedure to be recorded. Two additional cameras in the corners of the room provided backup. Prior to brushing with the BV1 participants were asked to move the brush once along the upper jaw from the left posterior molar to the right posterior molar with one stroke. They also were individually fitted with a head frame equipped with magnets. These procedures served other features of the Brushalyze project described in detail in the Appendix. Apart from these special features, the rest of the process was exactly the same for both brushes. Toothpaste (Meridol, CP GABA, Hamburg, Germany), a water cup and water were provided. Participants were asked to brush to the best of their abilities (“Please brush your teeth as good as you can, so that they are completely clean”) with their assigned toothbrush and without a time limit. Video recording was started and participants were left alone. Immediately after brushing, participants underwent sham staining and sham assessment of plaque (sham rather than real staining was necessary to avoid visible staining influencing behaviour at the second visit). Finally, participants were asked to answer some questions on the tablet. First, they had to assess their self-perceived oral cleanliness by a visual analogue scale ranging from not at all clean to totally clean (SPOC_n_, [25]). Then, questions were presented about the toothbrush they had just used compared to the one they used at home in terms of handling, stiffness of the bristles and weight, and finally, age and gender were asked.

*Second appointment* (one week later): CB welcomed the participant and NB assessed papillary bleeding. Then they were asked to brush their teeth in exact the same manner like in the first session. But now participants brushed with the other brush. Immediately after brushing, plaque was assessed. The final task was again a tablet survey: The questionnaire started identically to the questionnaire of the first appointment (i.e. the assessment of the SPOC_n_ and the comparison of the toothbrush just used to the usual toothbrush). Then the participants had to compare the toothbrush they had just used with the toothbrush used at the first appointment (again in terms of handling, bristle stiffness and weight). Subsequently, the participants answered in more detail the self-perceived cleanliness after they had been informed about the way a dentist would assess it (SPOC_d_, [25]). The questionnaire ended with some open questions about the BV1, in which positive and negative aspects of the toothbrush itself and the head frame were to be mentioned.

### Behavioural outcomes: observed toothbrushing

Video anlayses were performed as decribed before by use of the software Mangold INTERACT® 18 and previously validated observation procedures (Mangold International, Arnsdorf, Germany) [26, 27]. Briefly, calibrated examiners (PE, TS and NB) assessed the *tooth contact time* (time during which the toothbrush touches the teeth, without interruptions such as spitting, rinsing, etc.), the *surface* (occlusal, inner, outer) brushed within this time and the *brushing movements* (a.o., horizontal, vertical, circular) that were carried out. The primary outcome variable was percentage tooth contact time on inner surfaces. Secondary outcomes were percentage tooth contact time on outer and on occlusal surfaces and percentage tooth contact time with circular and with vertical movements [28, 29]. Calibration began with written and oral instructions regarding the observation categories and the use of the observation software. Then, the examiners analysed sample videos from previous studies. The calibration criterion was an intra class correlation of ICCs ≥ 0.90 between the observer and the previous annotations for at least five consecutive videos. If the criterion was missed, the observer was reinstructed and analysed additional videos until the criterion was reached. To assess the quality of annotations after calibration, 10 videos were randomly selected for each behavioural category and double-anotated by another calibrated examiner (WP, TS); the agreement between the observers was excellent (all ICCs ≥ 0.90). All examiners besides NB were fully blinded regarding the clinical or questionnaire data. NB performed the annotation several weeks after the assessment of the clinical data (November 2022 ─ April 2023), in order to minimise bias due to knowledge of the clinical data.

### Clinical data

Clinical assessments were performed by a calibrated dentist (NB) who was blinded with respect to the participants' group membership, their oral hygiene behaviour and the questionnaire data. The calibration procedure was similar to previous studies [28, 30]: An experienced calibrated dentist (WP) instructed NB. Afterwards both assessed the same individuals independently. They did not reveal their scores until they had both finished with an individual. Calibration was considered successful if, for five consecutive individuals, at least 90% of the ratings were identical and the remainder differed by no more than one point.

A simplified version of the papillary bleeding index (PBI, [31]) was assessed to control for gingival health. Only the presence or absence of papillary bleeding was assessed. These parameters were assessed on all present teeth (including the third molars).

The DMFT-Index was assessed at the first clinical examination of the first appointment. Third molars were not included into that assessment in order to comply with the principles of the World Health Organisation [32].To test whether the handle affected plaque persistence plaque levels were assessed at the second appointment immediately after brushing after staining (Miradent Mira-2-Ton®; Miradent, Germany). The primary clinical outcome for this analysis was the Marginal Plaque Index (MPI, [33]); the MPI assesses whether plaque is present (= 1) or absent (= 0) at eight equally sized sections at the gingival margin (four each at the inner/outer gingival margin). As an additional parameter the more coronal parts of the crown as indicated by the percentage of scores 3–5 of the Turesky modification of the Quigley and Hein Index (TQHI, [34]) were assessed; score 3 describes a plaque band wider than 1 mm but covering less than 1/3 of the crown, score 4 plaque covering at least 1/3 but less than 2/3 of the crown and score 5 plaque covering at least two-thirds of the crown.

### Subjective evaluation of the brush

Within the current analysis the subjective evaluations of the brushes serve as a manipulation check. At the end of each appointment the participants answered Likert scales to evaluate the handling and the nature of the brush they just had used in comparison to the one they use at home. At the end of the second appointment they also answered items directly comparing both brushes (the questions of these questionnaires are shown in the Appendix). Additionally, self-perceived oral cleanliness (SPOC) was assessed by a validated questionnaire [25]. Participants provided their naïve SPOC-estimation (SPOC_n_) after both appointments and their SPOC-estimation according to the standards of a dentist (SPOC_d_) after the second appointment only. For assessment of SPOC_d_ participants receive information how the MPI is assessed. Afterwards they estimate in 12 regions how many sections would be free of plaque. This is summarised in a score that can be read as an inverted MPI, as it indicates the percentage of (self-perceived) cleanliness rather than the percentage of plaque. The primary outcome regarding handling was the direct comparison of the two brushes regarding handling. The secondary outcome was SPOC_d_ and SPOC_n_ and the indirect comparison of the handling (i.e. the comparison of the evaluations with respect to the tooth brush used at home). The primary outcome regarding the nature of the brush was the direct comparison of the bristle stiffness, the secondary outcome was the indirect comparison of bristle stiffness and the direct comparison of the weight.

### Statistics

The research hypothesis stated that the brush would influence the extent to which certain behavioural parameters are displayed, but not the position of the person in the group. It was thus expected that the means would differ, but that the correlations between repeated measures would be high. Correlations (r) are interpreted in analogy to Guilford [35]: a high correlation and marked relationship lies between |.70| and |.90|, correlations above |.90| are described as very high and as a very dependent relationship. According to Cohen [36], standardized mean differences (SMD) of d ≥|.2| |.5| |.8| are considered small, medium and large, respectively. This was translated into the following statistical hypotheses pairs: H_0_: µ_BV1_ = µ_MT_; H_1_: µ_BV1_ ≠ µ_MT_ and H_0_: ρ_BV1,MT_ ≤ 0.5; H_1_: ρ_BV1,MT_ > 0.5. Power calculation with G*Power [37] indicated that a sample size of 50 would allow for the detection of small to medium SMD and correlations of ρ > 0.72 with alpha = 0.05 and a power of 1—β = 0.80.

Statistical procedures were performed using IBM SPSS Statistics, Version 29.0 (IBM SPSS Statistics for Windows; IBM, Armonk, New York, USA). Correlations are reported as Pearson and as Spearman correlations since single outlying values can distort results of Pearson correlations; 95% confidence intervals (95% CI) are reported along with Pearson correlations. T-Tests for dependent variables are computed to test for equality of the means of repeated measures. For behavioral analysis additional Wilcoxon tests are computed to control for distortions due to outlying values. T-tests for independent variables are used to compare baseline values and the means of variables assessed at the second appointment, only (plaque, SPOC_d_).

## Results

### Description of the sample

Figure 1 of the appendix shows the participant flow. Fifty participants (18 m, 32 f) aged 18–34 years (26.8 ± 4.1) completed data assessment. DMFT varied from 0–16 (5.2 ± 4.4), 37 participants had no decayed teeth, 11 had no filled teeth and 39 had no missing teeth. Nearly half of the papillae showed papillary bleeding at the first appointment (45.3% ± 18.2). No intraindividual differences were observed regarding papillary bleeding at the first and the second appointment (t(49) = 0.42; *p* = 0.68). Participants of the two sequences of brushing (BV1-MT vs. MT-BV1) did not differ regarding any of the above mentioned variables (all t(48) < 1.76, all *p* > 0.08).

### Manipulation check

**Fig. 2:**
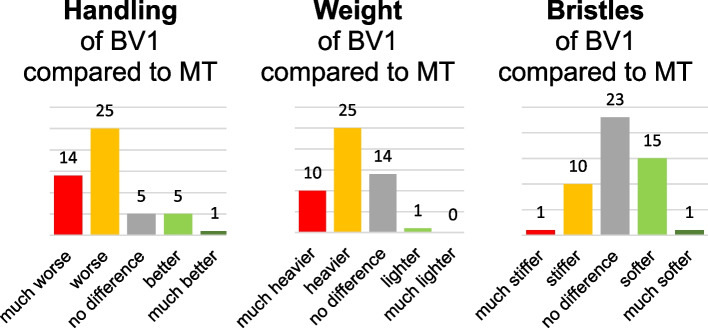
Distribution of answers given by participants when comparing the BV1 with the MT at the second appointment. The numbers above the bars indicate the number of people who rated the BV1 in this way

When participants directly compared the handling of the two brushes at the second appointment, the majority judged the handling of BV1 as being worse or much worse than that of the MT (see Fig. 2). The indirect comparison revealed a significant difference in this regard favouring the handling of MT (t(49) = 6.5; *p* < 0.01; d = 0.91). Also, the evaluation of the open questions shows that *n* = 38 participants named negative aspects of the BV1, which mainly comprised the handling and shape of the handle. Hence *n* = 22 recommended to improve the ergonomics of the handle. This did not go along with a difference regarding SPOC_d_ at the second appointment (t(48) = 0.085; *p* = 0.93; d = 0.024) or SPOC_n_ assessed immediately after brushing with the respective brush (t(49) = 0.934; *p* = 0.355; d = 0.132). SPOC_d_ scores indicate that participants estimated that 69% of gingival margin sections would be free of plaque deposits (BV1: 69.21 ± 13.61; M1: 69.54 ± 14.27).

Regarding weight, the majority of the participants rated the BV1 as being heavier than the MT in the direct comparison (see Fig. 2). Likewise, the indirect comparison revealed a significant difference in a sense that BV1 was judged as being heavier than MT (t(49) = 6.4; *p* < 0.001; d = 0.91). The indirect comparison regarding bristle stiffness revealed no difference between the two brushes (t(49) = 0.76; *p* = 0.45; d = 0.11), and the results of the direct comparison are distributed symmetrically around the most frequently chosen option “no difference” (see Fig. 2).

### Brushing behaviour

Table 1 shows results of behavioural analyses when participants brushed with the BV1 vs. MT. Regarding the primary outcome, no statistical difference of the means was found (d = 0.23; *p* = 0.11) but a significant correlation (*r* = 0.780; *p* < 0.05). Further comparisons of means reveal the largest difference for brushing time at inner surfaces (d = 0.31) and the time by which inner surfaces were brushed by horizontal movements (d = 0.35). The lowest correlation between the behavioural parameters was observed with regard to the percentage of time distributed to brushing outer surfaces (*r* = 0.72). All other correlations were higher, some of them exceeded *r* = 0.90 (see Table 1).

**Table 1:** Behavioural data

**Primary outcome**	MT		BV1		Comparison of means			Correlations	
	M	SD	M	SD	t	d	*p*	r*	rho
Inner surfaces (%tct)	22.56	(12.11)	20.77	(11.25)	1.63	0.23	0.11	0.780	0.733
**Secondary outcomes**									
Tooth conctact time (s)	211.87	(87.46)	209.36	(84.29)	0.47	0.07	0.64	0.904	0.901
Outer surfaces (%tct)	48.37	(11.04)	49.08	(12.01)	-0.59	-0.08	0.56	0.724	0.667
Occlusal surfaces (%tct)	29.07	(13.13)	30.15	(13.43)	-0.85	-0.12	0.40	0.770	0.769
Circular brushing on outer surfaces (%)	57.71	(29.54)	55.95	(27.41)	0.91	0.13	0.37	0.887	0.832
Vertical brushing on inner surfaces (%)	27.04	(29.17)	31.59	(30.90)	-1.83	-0.26	0.07^+^	0.833	0.830
**Further behavioural data**									
Inner surfaces (s)	51.04	(35.35)	45.09	(31.61)	2.21	0.31	0.03	0.843	0.804
Vertical brushing on inner surfaces (s)	16.42	(21.65)	16.55	(21.24)	-0.10	-0.01	0.92	0.901	0.868
Horizontal brushing on inner surfaces (s)	33.06	(26.85)	29.96	(21.24)	2.50	0.35	0.02	0.766	0.778
Horizontal brushing on inner surfaces (%)	69.95	(29.24)	66.12	(31.51)	1.49	0.22	0.14	0.821	0.762
Outer surfaces (s)	100.16	(41.32)	100.79	(41.92)	-0.16	-0.02	0.87	0.786	0.768
Circular brushing on outer surfaces (s)	58.07	(40.08)	58.00	(40.34)	0.03	< 0.01	0.98	0.883	0.850
Horizontal brushing on outer surfaces (s)	32.87	(36.12)	34.43	(32.37)	-0.76	-0.11	0.45	0.915	0.831
Horizontal brushing on outer surfaces (%)	33.87	(29.51)	35.43	(28.14)	-0.87	-0.12	0.39	0.903	0.846
Occlusal surfaces (s)	60.67	(37.14)	63.48	(41.69)	-1.00	-0.14	0.32	0.880	0.812

MT: commercially available manual brush; *BV1* Brushalyze Version 1, *tct* Tooth contact time; *all *p *≤ 0.05; all 95%CI *r* ≥ 0.58─*r* = 1; ^+^exact *p* < 0.05 in Wilcoxon-test

### Plaque after brushing

Plaque was assessed at the second appointment only. Those, who brushed with BV1 showed an MPI of 79.69 ± 11.08, indicating that nearly 80% of the assessed sections of the gingival margin showed persistent plaque. Those who brushed with MT had a mean MPI of 74.53 ± 11.6 (t(48) = 1.61; *p* = 0.115; d = 0.454). Regarding plaque on the more coronal parts of the crown (TQHI 3–5), BV1 revealed 55.22 ± 14.68 percent surfaces with plaque and MT 46.05 ± 16.2 percent (t(48) = 2.094; *p* = 0.042; d = 0.592).

## Discussion

The aim of the present study was to examine the impact of the toothbrush handle on toothbrushing behaviour. For this purpose, two toothbrushes with widely differing handles were contrasted: One was a conventional manual toothbrush with an ergonomic handle (MT) and the other was a toothbrush with a thick cylindrical handle with hardly any ergonomic features (BV1). In terms of the manipulation check, the results show that the selected toothbrushes were actually perceived differently by the participants. As intended, this dissimilarity concerned the perception of the handle and the weight of the brush but not the perception of the bristles. However, although the participants found the BV1 more unhandy, their self-perceived oral cleanliness was not affected by the brushes. Regarding objective plaque levels, there was in fact no difference observed at the gingival margins. Only the more coronal parts of the showed slightly less persistent plaque after the MT as compared to the BV1. These findings confirm previous research in two respects. Firstly, individuals significantly overestimate their oral cleanliness. They believe that they have cleaned 70─80% of the sites measured at the gingival margins [25, 38]. In fact, they only achieve a cleanliness of 30─40% ─ regardless of whether they brush to the best of their abilities or as usually [28, 29, 38, 39]. Secondly, this study confirms findings indicating that the handle plays a subordinate role in the removal of plaque [13, 40–42].

The research hypothesis of this study suggested that the type of toothbrush (BV1 vs. MT) would affect the extent to which certain behavioural parameters are displayed, but not the position of the person within the group. It was therefore expected that the mean values of the behavioural parameters would differ, but that the correlations between the repeated measures would be high. The results in Table 1, however, are not in support of the first part of this hypothesis. The null-hypothesis that states that the means would not differ (H_0_: µ_BV1_ = µ_MT_) is retained. Percentage of brushing time at inner surfaces was considered the primary outcome. Numerous observational studies have shown, that brushing of inner surfaces is most frequently neglected. Thus, inner surface seem to be the most difficult to reach [26, 27, 29, 30, 38, 43, 44]. Accordingly, it was assumed that the handiness of the toothbrush would play an even more important role here than with the other behavioural variables. Yet, the results in Table 1 show that the brush did not influence the percentage of time by which participants brushed their inner surfaces. Merely when expressed in absolute figures there is a small decline in brushing time at inner surfaces with BV1. However, this is not reflected in the time taken to perform the more complex vertical movements. Instead it only goes ahead with a decrease in the time taken to clean with horizontal movements. Nevertheless, these differences are too small to result in significant differences in the percentage of time spent on these movements on inner surfaces. All other behavioural differences observed are even smaller than the one’s highlighted so far. Thus, neither formal statistical analysis nor descriptive analysis of all the behavioural data supports the hypothesis that the handle of the brush would have a relevant influence on the extent of the behavioural components observed. One should keep in mind, however, that this observation refers to a group of young adults. Results might differ in groups with limited dexterity like young children or the elderly. The present participants found brushing more cumbersome with BV1 but apparently were able to compensate for that and remained stable in their behaviour. People with limited dexterity might, however, lack the capacity to compensate for the ergonomic drawbacks of the BV1.

The second part of the hypothesis states that the position of a person in the group would remain fairly stable even if the brush handle changes. This would result in positive correlations between the two measurement times. The data are in support of this hypothesis and the respective null-hypothesis (H_0_: ρ_BV1,MT_ ≤ 0.5) is rejected (see Table 1). All but one of the parameters show high to very high correlations between measurement times. This finding is in line with previous findings that have investigated the stability of repeated brushing with the same brush [24]. It extends this finding in demonstrating, that even a considerable change of the ergonomics of the brush does not influence the individual pattern of the brushing behaviour.

This result is consistent with the assumption that, once established, tooth brushing is a routine, almost automatic behavior more or less performed subconsciously [45]. This would also explain why people have difficulties accepting or maintaining instructions from training or prophylaxis programmes on how to brush their teeth correctly [26–29]. Once a behaviour is habituated, it is very difficult to change it again [46, 47]. At the same time, knowledge about the stability of toothbrushing behaviour opens up new ways to improve individual toothbrushing behaviour by detecting specific individual deficits and retraining them in a targeted manner.

The current study has certain strengths. It is a randomised counterbalanced cross over study that allows clear causal inferences. The blinding of the investigators and participants, the high degree of standardisation and the careful training and calibration of the personnel also contributes to its validity. In order to keep the general conditions as constant as possible at the first and second appointment, only a sham staining and sham assessment of dental plaque was done at the first appointment. Otherwise, there was the risk that the participants would have changed their brushing at the second appointment due to a visible staining of the plaque. However, this and the highly standardised laboratory conditions limit the generalisability of the results. Additionally, the data presented here refer to young adults with no limitations regarding their manual dexterity. Future studies should analyse whether the results of this study also apply to other groups like children or the elderly. Recent data suggest that special needs groups show improvements in plaque when brushing with a customized handle [18]. It is therefore important to bear in mind that in groups other than those studied here, a non-ergonomic handle may strongly influence plaque removal. So far no data is available concerning the stability of tooth-brushing behaviour in daily life. For such an analysis, more automated assessment tools would be needed that allow behavioural assessment without disturbing the person in their daily routine. Such tools could also improve behavioural analysis itself. Video analysis, which forms the basis of the present study, has clear limitations. Both the assessment of the position of the brush and the pattern of movement are confined to rather broad categories. And even then, it takes 4–6 h to complete an analysis of one tooth-brushing process. Moreover, video analysis does not inform about the pressure applied with the brush, which is also an important parameter [48, 49]. The current study is part of a project that tries to overcome these limitations by developing an automated tool to assess tooth-brushing behaviour (DFG; Project number 448034414; https://gepris.dfg.de/gepris/projekt/448034414?language=en). The current study was also designed to provide information about the impact that such an automated tool would have on at least the broad categories of behaviour that can be assessed from video data.

## Conclusion

In summary, the results of this study indicate that the handle of a toothbrush does not play a significant role in toothbrushing behaviour in people whose dexterity is not limited. Rather, it seems to be the habit that determines the brushing behaviour.

### Supplementary Information

Supplementary Material 1.

## Data Availability

The datasets used and/or analyzed during the current study are available from the corresponding author on reasonable request. However, for privacy reasons, no individual data allowing identification of participants (e.g., videos) can be provided.

## Abbreviations

BV1: Brushalyze Version 1
CB: Carlotta Bottenbruch (author)
ICC: Intra class correlation
MPI: Marginal Plaque Index
MT: Commercially available manual toothbrush
NB: Nils Berneburg (author)
PBI: Papillary bleeding index
PE: Assistant1
SPOC: Self perceived oral cleanliness
TQHI: Turesky’s modification of the plaque index of Quigley and Hein
TS: Assistant2
WHO: World Health Organization
WP: Assistant3

## Footnotes

1 Purodent, Replacement brushes, medium (see also Fig. 1).

2 Oral-B toothbrush1,2,3 Indicator extra soft; Procter & Gamble Service, Schwalbach, Germany.

3 1) Dr. Best Classic, medium hard; GlaxoSmithKline Consumer Healthcare, Munich, Germany 2) Bamboo toothbrush Virgin Forest,, medium soft; The Bamboo Toothbrush Store, Canada 3) TIO Toothbrush with travel cap, medium hard; TIO, Berlin, Germany 4) Oral-B toothbrush 1,2,3 Indicator extra soft; Procter & Gamble Service, Schwalbach, Germany 5) Oral-B toothbrush 1,2,3 Indicator Medium/Medium; Procter & Gamble Service, Schwalbach, Germany.
