# Correction: It is the habit not the handle that affects tooth brushing - a randomised counterbalanced cross over study with young and healthy adults

**DOI:** 10.1186/s12903-024-04673-0

**Published:** 2024-08-02

**Authors:** Renate Deinzer, Zdenka Eidenhardt, Keywan Sohrabi, Manuel Stenger, Dominik Kraft, Bernhard Sick, Franz Götz‑ Hahn, Carlotta Bottenbruch, Nils Berneburg, Ulrike Weik

**Affiliations:** 1 Department of Medicine, Justus-Liebig-University Giessen, Klinikstr. 29, 35392 Giessen, Germany; 2 Faculty of Health Sciences, University of Applied Sciences Giessen, Ostanlage 45, 35390 Giessen, Germany; 3 Intelligent Embedded Systems, University of Kassel, Wilhelmshöher, Allee 73, 34121 Kassel, Germany

**Correction to: BMC Oral Health (2024) 24:757**

10.1186/s12903-024-04538-6

In this article [1], the affiliation details for the Author Manuel Stenger was incorrectly given as ‘Department of Medicine, Justus-Liebig-University Giessen, Klinikstr. 29, Giessen 35392, Germany’ but should have been ‘Faculty of Health Sciences, University of Applied Sciences Giessen, Ostanlage 45, Giessen 35390, Germany’.
