# Supplementary material for: It is the habit not the handle that affects tooth brushing - a randomised counterbalanced cross over study with young and healthy adults
Source: BMC Oral Health. 2024 Jul 2;24:757. doi: 10.1186/s12903-024-04538-6 (PMC11218271; doi:10.1186/s12903-024-04538-6)
Supplement: Supplementary file 1 — Supplementary Material 1. [file 12903_2024_4538_MOESM1_ESM.pdf]

Appendix

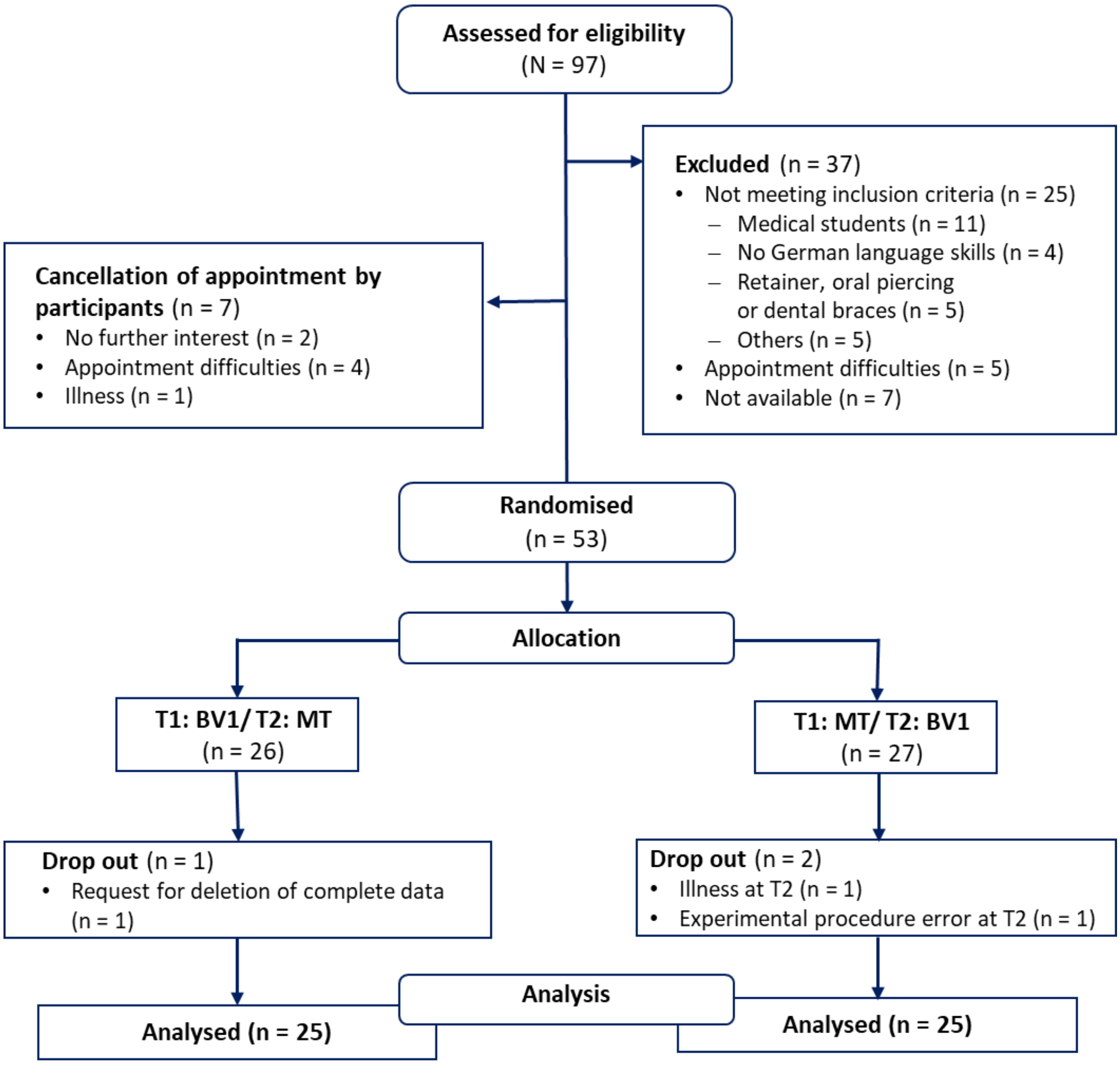

Fig. 1 Flowchart of the recruitment, randomisation and analysis

Description of the Brushalyze Project

“*Brushalyze - Understanding the tooth brushing process all along: New research device for multi-sensorial detection and intelligent analysis of tooth brushing*”. (Deutsche Forschungsgemeinschaft (DFG) – Project number 448034414; https://gepris.dfg.de/gepris/projekt/448034414?language=en)

Developed as part of the innovative Brushalyze project, the "Brushalyze-V1" toothbrush aims to redefine traditional dental hygiene research methodology by introducing a new paradigm for comprehensive, three-dimensional monitoring of toothbrushing activities. The embedded suite of sophisticated sensors within the handle yields data that encompasses not only the spatial position and dynamic movement of the toothbrush within a 3D space. Participants also wear external wear sensors when using the brush to better determine the exact position of the brush. In addition, pressure-sensitive behaviors upon tooth surfaces are meticulously scrutinized. Inherently, this detailed motion analysis propels the investigative lens beyond the confines of previous methodologies, such as time-consuming video analysis of brushing behaviour.

The data, harvested from the embedded sensors, will become instrumental in crafting algorithms. These are engineered to pinpoint not only the precise position of the brush during its utilization but also to decode specific motion patterns and pressure distributions throughout the brushing process. Moreover, the incorporated technology within "Brushalyze-V1" facilitates a sensor-based AI evaluation of the collected data, thereby embedding an intelligent, analytical layer to brushing analytics. This advancement in sensor-driven AI evaluation aims to improve research on oral hygiene practices through data-driven insights. The data from the current project will therefore also feed into the development of the algorithms needed to evaluate the sensor data.

Questionnaires

A translation of the questions into English is provided at the end of this document. The translation presented is intended to help international readers understand the structure and content of the questions. It is not intended as a scientifically validated translation that can be used in future research.

Questions at the first appointment

JUSTUS-LIEBIG-UNIVERSITÄT GIESSEN

17% ausgefüllt

Im Folgenden finden Sie verschiedene Fragen zum Zähneputzen und zu Ihrer Person.

Beachten Sie bitte: Es gibt **keine richtigen oder falschen Antworten** und **keine guten oder schlechten**.

Wichtig ist, dass Sie auswählen, was für Sie am besten zutrifft.

Weiter

33% ausgefüllt

Erfassung des SPOC$_n$

[Der SPOC-Fragebogen findet sich im Anhang der Publikation: Eidenhardt Z, Busse S, Margraf-Stiksrud J, Deinzer R. Patients' awareness regarding the quality of their oral hygiene: development and validation of a new measurement instrument. BMC Oral Healh. 2022; 22:629. doi:10.1186/s12903-022-02659-4.]

Weiter

50% ausgefüllt

Im Folgenden möchten wir Sie darum bitten, die **soeben genutzte** Zahnbürste mit der zu vergleichen,

die Sie **normalerweise daheim** verwenden.

Weiter

67% ausgefüllt

Bitte beantworten Sie die folgenden Fragen.

Uns interessiert Ihre Einschätzung. Klicken Sie das Feld an, das Ihrer Meinung am ehesten entspricht.

Wie empfanden Sie die Handhabung dieser Zahnbürste im Vergleich zu Ihrer gewohnten Zahnbürste?

○	○	○	○	○
viel unhandlicher	unhandlicher	kein Unterschied	handlicher	viel handlicher

Wie empfanden Sie die Härte der Borsten dieser Zahnbürste im Vergleich zu Ihrer gewohnten Zahnbürste?

○	○	○	○	○
viel härter	härter	kein Unterschied	weicher	viel weicher

Wie empfanden Sie das Gewicht dieser Zahnbürste im Vergleich zu Ihrer gewohnten Zahnbürste?

○	○	○	○	○
viel schwerer	schwerer	kein Unterschied	leichter	viel leichter

Weiter

83% ausgefüllt

Wie alt sind Sie?

[ ] Jahre

Welches Geschlecht haben Sie?

- ○ männlich
- ○ weiblich
- ○ anderes

Welches ist Ihr höchster Bildungsabschluss?

[Bitte auswählen]

Weiter

Vielen Dank - Sie haben das Ende des Fragebogens erreicht.

Bitte geben Sie uns Bescheid, dass Sie die Befragung abgeschlossen haben.

Questions at the second appointment

JUSTUS-LIEBIG-UNIVERSITÄT GIESSEN

0% ausgefüllt

Wie bereits beim letzten Termin finden Sie im Folgenden wieder verschiedene Fragen rund um das Thema Zähneputzen.

Beachten Sie bitte: Es gibt **keine richtigen oder falschen Antworten** und **keine guten oder schlechten**.

Wichtig ist, dass Sie auswählen, was für Sie am besten zutrifft.

Weiter

4% ausgefüllt

Erfassung des $SPOC_n$

Die konkrete Abfrage des SPOC findet sich im Anhang der Publikation: Eidenhardt Z, Busse S, Margraf-Stiksrud J, Deinzer R. Patients' awareness regarding the quality of their oral hygiene: development and validation of a new measurement instrument. BMC Oral Healh. 2022; 22:629. doi:10.1186/s12903-022-02659-4.

Weiter

4% ausgefüllt

Im Folgenden möchten wir Sie darum bitten, die **soeben genutzte** Zahnbürste mit der zu vergleichen,

die Sie **normalerweise daheim** verwenden.

Weiter

9% ausgefüllt

Bitte beantworten Sie folgende Fragen.

Uns interessiert Ihre Einschätzung. Klicken Sie das Feld an, das Ihrer Meinung am ehesten entspricht.

Wie empfanden Sie die Handhabung dieser Zahnbürste im Vergleich zu Ihrer gewohnten Zahnbürste?

○	○	○	○	○
viel unhandlicher	unhandlicher	kein Unterschied	handlicher	viel handlicher

Wie empfanden Sie die Härte der Borsten dieser Zahnbürste im Vergleich zu Ihrer gewohnten Zahnbürste?

○	○	○	○	○
viel härter	härter	kein Unterschied	weicher	viel weicher

Wie empfanden Sie das Gewicht dieser Zahnbürste im Vergleich zu Ihrer gewohnten Zahnbürste?

○	○	○	○	○
viel schwerer	schwerer	kein Unterschied	leichter	viel leichter

Weiter

11% ausgefüllt

Im Folgenden möchten wir Sie darum bitten, die **soeben genutzte** Zahnbürste mit der zu vergleichen,

die Sie **beim ersten Untersuchungstermin** verwendet haben.

Weiter

13% ausgefüllt

"Im Vergleich zur Zahnbürste, die ich beim ersten Termin verwendet habe, fand ich ..."

... die <u>Handhabung</u> der heute verwendeten Zahnbürste gegenüber der vom ersten Termin ...

○	○	○	○	○
viel unhandlicher	unhandlicher	kein Unterschied	handlicher	viel handlicher

... die <u>Borsten</u> der heute verwendeten Zahnbürste gegenüber der vom ersten Termin ...

○	○	○	○	○
viel härter	härter	kein Unterschied	weicher	viel weicher

... das <u>Gewicht</u> der heute verwendeten Zahnbürste gegenüber der vom ersten Termin ...

○	○	○	○	○
viel schwerer	schwerer	kein Unterschied	leichter	viel leichter

Weiter

15% ausgefüllt

Wie bereits bei der ersten Frage finden Sie im Folgenden wieder verschiedene Fragen rund um das Thema Zähneputzen.

Beachten Sie bitte weiterhin: Es gibt **keine richtigen oder falschen Antworten** und **keine guten oder schlechten**.

Wichtig ist, dass Sie auswählen, was für Sie am besten zutrifft.

Weiter

17% ausgefüllt

Erfassung des SPOC$_d$

Der SPOC-Fragebogen findet sich im Anhang der Publikation: Eidenhardt Z, Busse S, Margraf-Stiksrud J, Deinzer R. Patients' awareness regarding the quality of their oral hygiene: development and validation of a new measurement instrument. BMC Oral Healh. 2022; 22:629. doi:10.1186/s12903-022-02659-4.

Weiter

67% ausgefüllt

Nun haben wir noch ein paar Fragen speziell zu dieser Bürste

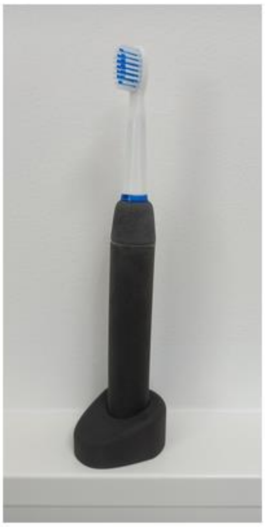

Weiter

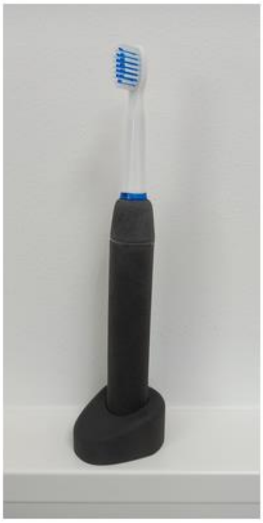

Sind Ihnen bei dieser Bürste bestimmte Aspekte positiv aufgefallen?

- (•) ja
- ( ) nein

Weiter

This question is only displayed if the previous answer was "Yes".

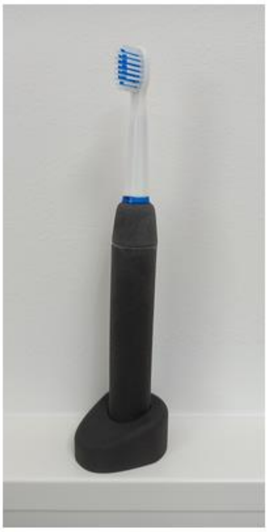

Welche Aspekte sind Ihnen bei dieser Bürste positiv aufgefallen?

Weiter

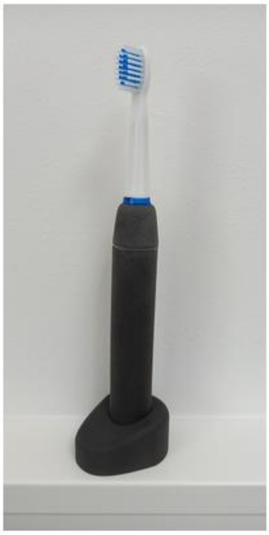

Sind Ihnen bei dieser Bürste bestimmte Aspekte negativ aufgefallen?

- ja
- nein

Weiter

This question is only displayed if the previous answer was "Yes".

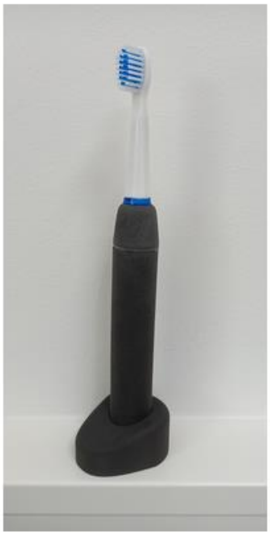

Welche Aspekte sind Ihnen bei dieser Bürste negativ aufgefallen?

Weiter

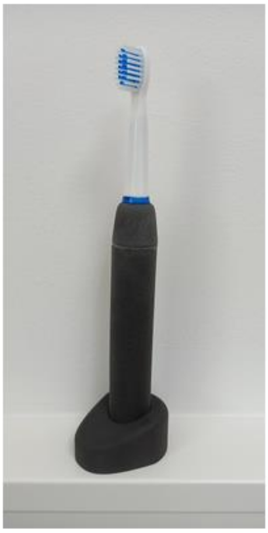

Haben Sie Anregungen oder Wünsche an uns, um diese Bürste zu verbessern?

- (•) ja
- ( ) nein

Weiter

This question is only displayed if the previous answer was "Yes".

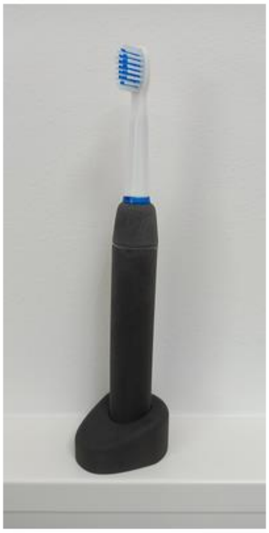

Was sollte an der Bürste verbessert werden?

Weiter

Abschließend haben wir noch einige Fragen zu dem verwendeten Kopfaufsatz

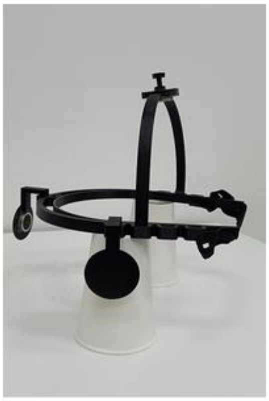

Weiter

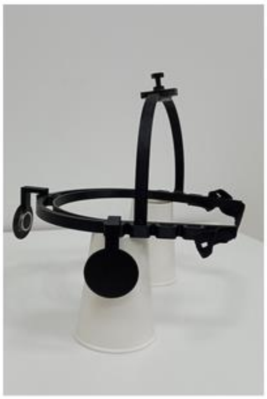

Sind Ihnen bei diesem Kopfaufsatz bestimmte Aspekte positiv aufgefallen?

- (●) ja
- ( ) nein

Weiter

This question is only displayed if the previous answer was "Yes".

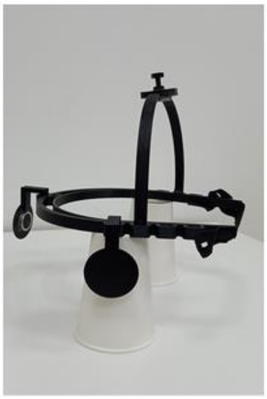

Welche Aspekte sind Ihnen bei dem Kopfaufsatz positiv aufgefallen?

Weiter

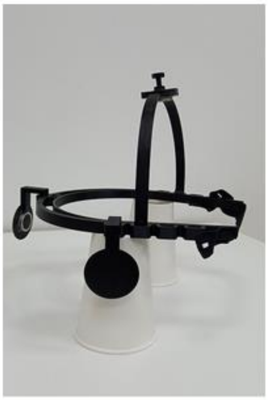

Sind Ihnen bei diesem Kopfaufsatz bestimmte Aspekte negativ aufgefallen?

- (x) ja
- ( ) nein

Weiter

This question is only displayed if the previous answer was "Yes".

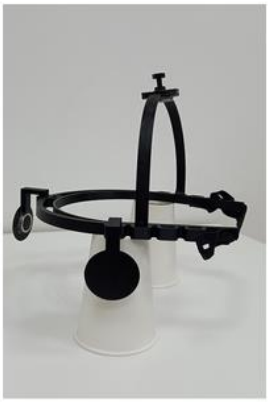

Welche Aspekte sind Ihnen bei dem Kopfaufsatz negativ aufgefallen?

Weiter

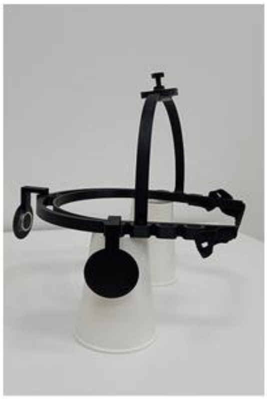

Haben Sie Anregungen oder Wünsche an uns, um den Kopfaufsatz zu verbessern?

- (•) ja
- ( ) nein

Weiter

This question is only displayed if the previous answer was "Yes".

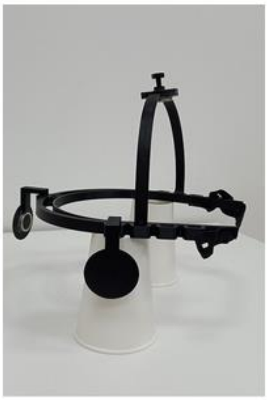

Was sollte am Kopfaufsatz verbessert werden?

[ ]

Weiter

Vielen Dank für Ihre Teilnahme!

Wir möchten uns ganz herzlich für Ihre Mithilfe bedanken.

Bitte geben Sie uns Bescheid, dass Sie die Befragung abgeschlossen haben.

Translated with the help of DeepL.
Visit www.DeepL.com/pro for more Information.

Questions at the first appointment

JUSTUS-LIEBIG-UNIVERSITÄT GIESSEN

17% ausgefüllt

In the following, you will find various questions about brushing your teeth and about yourself.

Please note: There are **no right or wrong answers** and **no good or bad ones**.

What is important is that you choose what applies best to you.

continue

33% ausgefüllt

Query of SPOC$_n$

The actual query of the SPOC can be found in the appendix of the publication: Eidenhardt Z, Busse S, Margraf-Stiksrud J, Deinzer R. Patients' awareness regarding the quality of their oral hygiene: development and validation of a new measurement instrument. BMC Oral Healh. 2022; 22:629. doi:10.1186/s12903-022-02659-4.

continue

This is a curtesy translation from German. A scientific translation protocol was not yet applied.

4% ausgefüllt

In the following, we would like to ask you to compare the toothbrush you **have just used** with the one that you **normally** use **at home.**

continue

67% ausgefüllt

Please answer the following questions.

We are interested in your assessment. Click the box that most closely matches your opinion.

How did you find the handling of this toothbrush compared to your usual toothbrush?

○	○	○	○	○
much more unhandy	more unhandy	no difference	more manageable	much more manageable

How did you find the stiffness of the bristles of this toothbrush compared to your usual toothbrush?

○	○	○	○	○
much harder	harder	no difference	softer	much softer

How did you feel about the weight of this toothbrush compared to your usual toothbrush?

○	○	○	○	○
much heavier	heavier	no difference	lighter	much lighter

continue

This is a curtesy translation from German. A scientific translation protocol was not yet applied.

83% ausgefüllt

How old are you?

[ ] years

Which gender do you have?

- ○ male
- ○ female
- ○ other

What is your highest level of education?

[please select]

continue

Thank you - you have reached the end of the questionnaire.

Please let us know that you have completed the survey.

This is a curtesy translation from German. A scientific translation protocol was not yet applied.

Questions at the <u>second</u> appointment

JUSTUS-LIEBIG-UNIVERSITÄT GIESSEN

0% ausgefüllt

As at the last appointment, you will again find various questions about brushing your teeth below.

Please note: There are **no right or wrong** answers and **no good or bad ones**.

What is important is that you choose what applies best to you.

continue

4% ausgefüllt

Query of SPOC$_n$

The SPOC-questionnaire is available in the appendix of the publication: Eidenhardt Z, Busse S, Margraf-Stiksrud J, Deinzer R. Patients' awareness regarding the quality of their oral hygiene: development and validation of a new measurement instrument. BMC Oral Healh. 2022; 22:629. doi:10.1186/s12903-022-02659-4.

continue

4% ausgefüllt

In the following, we would like to ask you to compare the toothbrush you **have just used** with the one that you **normally** use **at home.**

continue

This is a curtesy translation from German. A scientific translation protocol was not yet applied.

9% ausgefüllt

Please answer the following questions.

We are interested in your assessment. Click the box that most closely matches your opinion.

How did you find the handling of this toothbrush compared to your usual toothbrush?

much more unhandy	more unhandy	no difference	more manageable	much more manageable
○	○	○	○	○

How did you find the stiffness of the bristles of this toothbrush compared to your usual toothbrush?

much harder	harder	no difference	softer	much softer
○	○	○	○	○

How did you feel about the weight of this toothbrush compared to your usual toothbrush?

much heavier	heavier	no difference	lighter	much lighter
○	○	○	○	○

continue

11% ausgefüllt

In the following, we would like to ask you to compare the toothbrush you **have just used** with the one that you used **at the first appointment**.

continue

This is a curtesy translation from German. A scientific translation protocol was not yet applied.

13% ausgefüllt

"Compared to the toothbrush I used at the first appointment, I found ..."

... the handling of the toothbrush used today compared to the one used at the first appointment ...

much more unhandy	more unhandy	no difference	more manageable	much more manageable
○	○	○	○	○

... the bristles of the toothbrush used today compared to the one used at the first appointment ...

much harder	harder	no difference	softer	much softer
○	○	○	○	○

... the weight of the toothbrush used today compared to the one used at the first appointment ...

much heavier	heavier	no difference	lighter	much lighter
○	○	○	○	○

continue

15% ausgefüllt

As with the first question, you will find various questions about brushing your teeth below.

Please note that there are **no right or wrong** answers and **no good or bad ones**.

What is important is that you choose what applies best to you.

continue

17% ausgefüllt

Query of SPOC$_d$

The SPOC-questionnaire is available in the appendix of the publication: Eidenhardt Z, Busse S, Margraf-Stiksrud J, Deinzer R. Patients' awareness regarding the quality of their oral hygiene: development and validation of a new measurement instrument. BMC Oral Health. 2022; 22:629. doi:10.1186/s12903-022-02659-4.

continue

This is a curtesy translation from German. A scientific translation protocol was not yet applied.

67% ausgefüllt

Now we have a few questions specifically about this brush

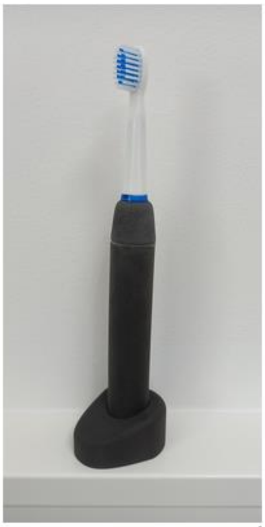

continue

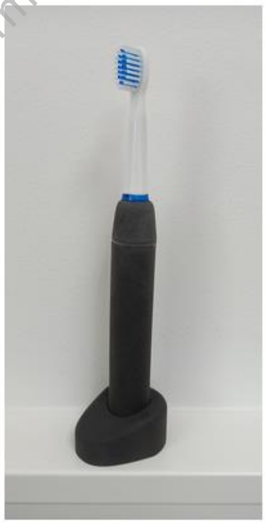

Did you notice any <u>positive</u> aspects of this brush?

- (•) yes
- ( ) no

continue

This is a curtesy translation from German. A scientific translation protocol was not yet applied.

This question is only displayed if the previous answer was "Yes".

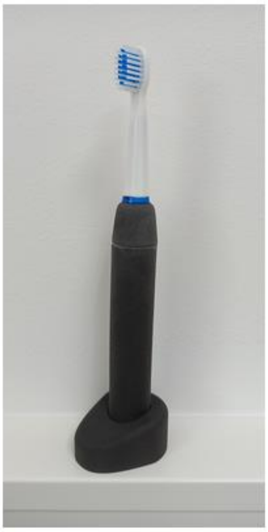

What aspects of this brush did you notice positively?

[ ]

continue

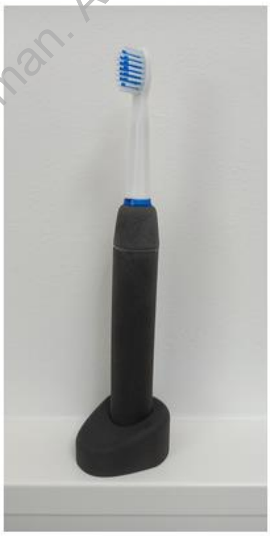

Did you notice any <u>negative</u> aspects of this brush?

- (•) yes
- ( ) no

continue

This is a curtesy translation from German. A scientific translation protocol was not yet applied.

This question is only displayed if the previous answer was "Yes".

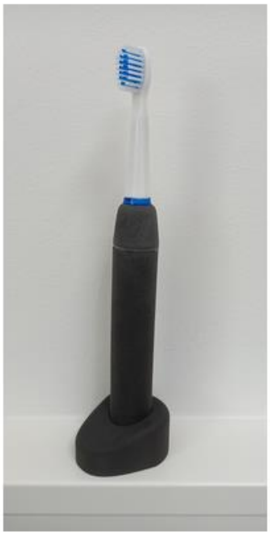

What aspects of this brush did you notice negatively?

continue

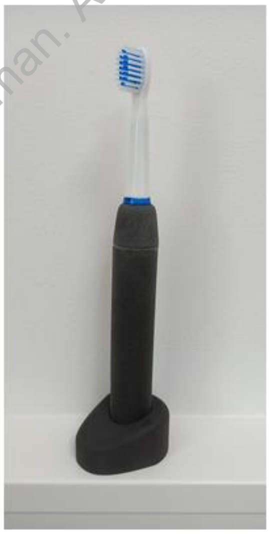

Do you have any suggestions or requests for us to improve this brush?

- yes
- no

continue

This is a curtesy translation from German. A scientific translation protocol was not yet applied.

This question is only displayed if the previous answer was "Yes".

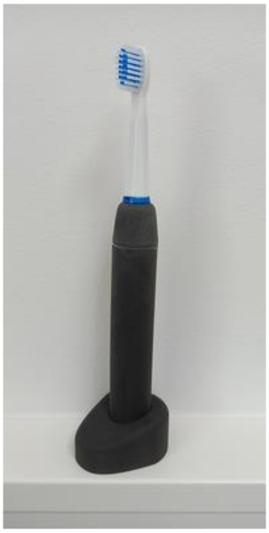

What should be improved about the brush?

continue

Finally, we have some questions about the head attachment used

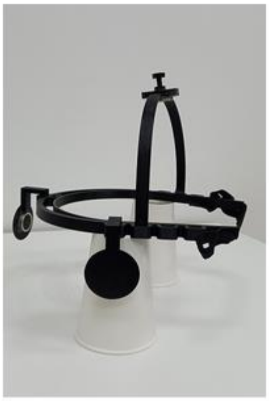

continue

This is a curtesy translation from German. A scientific translation protocol was not yet applied.

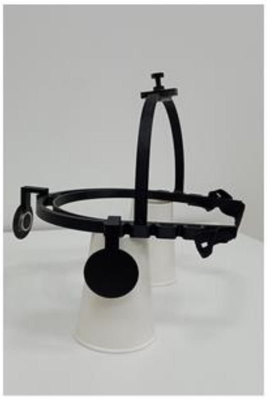

Did you notice any positive aspects of this head attachment?

- (•) yes
- ( ) no

continue

This question is only displayed if the previous answer was "Yes".

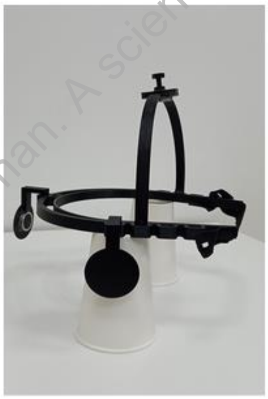

What aspects of the head attachment did you notice positively?

continue

This is a curtesy translation from German. A scientific translation protocol was not yet applied.

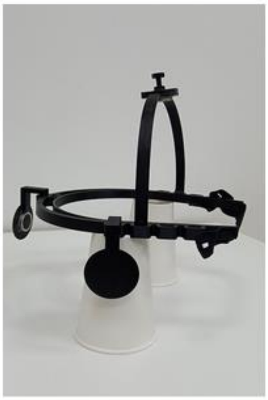

Did you notice any negative aspects of this head attachment?

- (•) yes
- ( ) no

continue

This question is only displayed if the previous answer was "Yes".

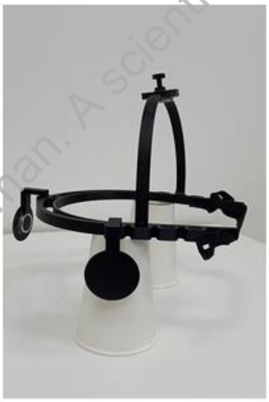

What aspects of the head attachment did you notice negatively?

continue

This is a curtesy translation from German. A scientific translation protocol was not yet applied.

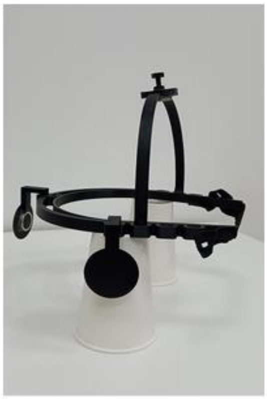

Do you have any suggestions or requests for us to improve the head attachment?

- [x] yes
- [ ] no

continue

This question is only displayed if the previous answer was "Yes".

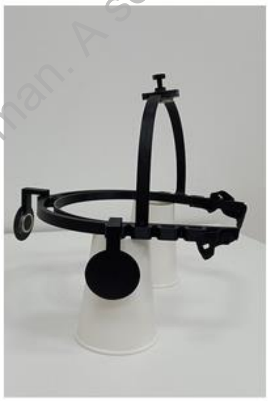

What should be improved about the head attachment?

continue

This is a courtesy translation from German. A scientific translation protocol was not yet applied.

Thank you very much for your participation!

We would like to thank you very much for your cooperation.

Please let us know that you have completed the survey.

This is a curtesy translation from German. A scientific translation protocol was not yet applied.
